# Oral Inflammatory Lesions and Bone Turnover Biomarkers (Dkk-1 and TRAP-5B) in Patients with ENT Cancer: A Radiological and Clinical Case–Control Study

**DOI:** 10.3390/medicina62040669

**Published:** 2026-04-01

**Authors:** Cristina Antohi, Eduard Radu Cernei, Sorina Solomon, Alexandra Corina Concita, Roxana Mihaela Popescu, Mihaela Salceanu

**Affiliations:** 1 Department of Odontology-Periodontology-Fixed Prosthetics, Faculty of Dental Medicine, Grigore T. Popa University of Medicine and Pharmacy Iasi, 16 Universitatii Street, 700115 Iasi, Romania; crisantohi_med@yahoo.com (C.A.); sorina.solomon@umfiasi.ro (S.S.); corina_alexandra2007@yahoo.com (A.C.C.); salceanu.mihaela@yahoo.com (M.S.); 2 Department of Oral and Maxillofacial Surgery, Faculty of Dental Medicine, Grigore T. Popa University of Medicine and Pharmacy Iasi, 16 Universitatii Street, 700115 Iasi, Romania

**Keywords:** periodontal pockets, dental caries, periapical lesions, cone beam computed tomography, Dickkopf-1, TRAP-5, ENT cancer

## Abstract

*Background and Objectives*: This study aimed to evaluate associations between dental caries, periodontal pockets, and radiologically detected periapical lesions in relation to serum levels of Dickkopf-1 (Dkk-1) and tartrate-resistant acid phosphatase 5B (TRAP-5B) in oncologic patients with ear, nose, and throat (ENT) cancer compared with healthy controls. *Materials and Methods*: The study included 63 subjects divided into a study group of 33 patients diagnosed with ENT cancer and a control group of 30 healthy individuals. Blood samples were collected to assess serum Dkk-1 levels using a sandwich enzyme immunoassay and TRAP-5B levels. Radiological dental evaluation included orthopantomography (OPT) and cone beam computed tomography (CBCT) to assess the number and depth of dental caries and the presence of periapical lesions. Periodontal pockets were recorded through clinical examination. *Results*: Serum biomarker analysis demonstrated significant differences between groups: TRAP-5B levels were significantly higher in patients with ENT cancer, whereas Dkk-1 concentrations were significantly lower compared with healthy controls (*p* < 0.001). OPT revealed up to eight carious lesions in both groups. The mean number of carious lesions was higher in healthy subjects (2.97 ± 2.48) than in patients with ENT cancer (2.06 ± 2.29). CBCT evaluation revealed 0–8 carious lesions in healthy individuals and 0–6 lesions in patients with ENT cancer, with a significantly higher mean number of lesions in the control group (2.97 ± 2.48 vs. 1.85 ± 1.89). Periodontal pockets were more frequent in patients with ENT cancer (0.67 ± 1.32) than in controls (0.37 ± 0.81). OPT evaluation also showed a higher mean number of periapical lesions in patients with ENT cancer (0.82 ± 1.29) compared with controls (0.37 ± 0.67). CBCT examination demonstrated that the mean number of periapical lesions in patients with ENT cancer was more than twice that of the control group, although this difference did not reach statistical significance. *Conclusions*: Patients with ENT cancer exhibited significantly altered systemic bone turnover biomarker profiles, characterized by increased TRAP-5B and decreased Dkk-1 levels. Clinically, these patients also presented a higher prevalence of periodontal pockets and periapical lesions, whereas carious lesions were more frequently detected in healthy individuals. The combined radiological and biochemical findings contribute to a better understanding of oral–systemic interactions in oncologic patients and highlight the importance of comprehensive dental evaluation prior to oncologic therapy.

## 1. Introduction

Dental caries is a biofilm-mediated, diet-modulated, multifactorial, non-communicable disease characterized by progressive mineral loss of dental hard tissues [1,2,3]. Its development is influenced by biological, behavioral, psychosocial, and environmental factors, ultimately leading to structural destruction of enamel and dentin [4]. Accurate diagnosis—based on clinical and radiographic evaluation—is essential for appropriate management and long-term disease control [5,6]. Contemporary caries management integrates primary, secondary, and tertiary preventive strategies under a comprehensive, risk-based approach [4,7].

Saliva plays a central protective role in maintaining enamel integrity and regulating demineralization–remineralization dynamics [8,9,10,11]. Conditions such as polypharmacy, Sjögren’s syndrome, and radiotherapy for head and neck cancer may impair salivary gland function, thereby increasing susceptibility to dental caries and dental erosion [12,13]. Dental caries and periodontitis represent the leading causes of tooth loss worldwide and share several behavioral and socioeconomic risk factors [14,15,16,17,18,19,20,21]. However, their potential biological interrelationships and systemic implications remain incompletely elucidated [21,22,23,24].

Radiographic assessment is fundamental in detecting proximal and periapical pathology. Conventional bitewing radiographs identify approximately 60% of proximal lesions [25,26,27,28], while digital systems have not substantially improved detection rates [29,30,31,32,33,34,35,36]. Panoramic radiography provides broad anatomical visualization with greater patient comfort [29], whereas cone beam computed tomography (CBCT) allows three-dimensional evaluation and has demonstrated diagnostic performance comparable to intraoral radiography in non-restored teeth [29,37,38,39,40,41,42,43].

Beyond local clinical parameters, systemic biomarkers may offer insight into oral–systemic interactions. Dickkopf-1 (Dkk-1), a Wnt signaling pathway inhibitor, regulates osteoblast differentiation and bone remodeling, and its dysregulation has been associated with inflammatory and neoplastic bone processes [44,45,46,47,48,49]. Tartrate-resistant acid phosphatase 5B (TRAP-5B) reflects osteoclast number and activity and is considered a reliable marker of bone resorption [50]. Alterations in these biomarkers may indicate systemic bone remodeling imbalance in inflammatory and oncologic conditions.

Radiation-related caries represents a severe complication in patients undergoing radiotherapy for head and neck cancer, resulting from salivary dysfunction, microbiota changes, mucosal alterations, and impaired oral hygiene [51]. Given the complex interaction between local oral pathology, systemic inflammation, and bone metabolism, investigating bone turnover biomarkers in oncologic patients may provide additional understanding of oral–systemic crosstalk.

Therefore, the aim of this study was to evaluate the association between dental caries, periodontal parameters, and radiologically detected periapical lesions with serum levels of Dickkopf-1 (Dkk-1) and tartrate-resistant acid phosphatase 5B (TRAP-5B) in patients with ENT cancer compared with healthy controls. We hypothesized that oncologic patients would exhibit altered systemic bone remodeling biomarkers and different patterns of association with oral inflammatory lesions compared with healthy individuals.

## 2. Materials and Methods

### 2.1. Study Design and Sample Size

This case–control study evaluated the association between radiologically detected oral inflammatory lesions and systemic bone remodeling biomarkers in patients with head and neck (ENT) cancer.

A total of 63 participants were recruited between June and November 2021 from the Regional Institute of Oncology Iași and the “Grigore T. Popa” University of Medicine and Pharmacy of Iași. The study group included 33 patients with histopathologically and CT-confirmed head and neck cancer. The control group comprised 30 systemically healthy individuals.

To ensure methodological rigor and reproducibility, standardized eligibility criteria were applied for both study groups. The study group included patients with histopathologically and computed tomography (CT)-confirmed head and neck cancer who had not yet undergone oncologic treatment, while the control group consisted of systemically healthy individuals without a history of malignancy.

Sample size estimation was performed using G*Power software (version 3.1), and the detailed parameters of the power analysis are described in Section 2.5 (Statistical Analysis).

Examiner calibration was conducted prior to data collection to ensure consistency in clinical and radiological assessments. Measurement reliability was evaluated using the intraclass correlation coefficient (ICC), demonstrating high intra-examiner agreement for periodontal probing and radiographic interpretation.

The study was approved by the Ethics Committee of the “Grigore T. Popa” University of Medicine and Pharmacy of Iași (Approval No. 85/26.05.2021) and conducted in accordance with the Declaration of Helsinki. All participants provided written informed consent.

### 2.2. Eligibility Criteria

Study Group

The inclusion criteria for the study group were age between 24 and 80 years, histopathologically and computed tomography (CT)-confirmed head and neck cancer, absence of distant metastases, no prior oncologic therapy, and the presence of dental pathology. Exclusion criteria included the presence of metastases, previous or ongoing radiotherapy or chemotherapy, age below 24 or above 80 years, and absence of dental pathology.

Control Group

The control group included individuals aged between 24 and 80 years with no history of malignancy and with the presence of dental pathology. Exclusion criteria were systemic diseases, malignancy, pregnancy or lactation, antibiotic use within the previous 3 months, and age outside the defined limits.

### 2.3. Clinical and Radiological Assessment

All participants underwent standardized clinical and radiological examination.

Periodontal Evaluation

Periodontal pockets were assessed using a calibrated periodontal probe. The number of sites with probing depth ≥4 mm was recorded.

Examiner calibration was performed prior to the study on 10 patients. Intra-examiner reliability showed high agreement (intraclass correlation coefficient [ICC] = 0.89).

Periodontal assessment was performed according to established clinical parameters, including probing depth and clinical attachment evaluation, as described in previous studies investigating periodontal disease and inflammatory burden [52,53].

Radiological Evaluation

Orthopantomography (OPT) and cone beam computed tomography (CBCT) were performed. Carious lesions were measured using Romexis software (v4.4.2), recording the maximum lesion diameter. Periapical lesions were assessed on both imaging modalities.

Radiological parameters:

OPT: 70–74 kV; 10–12.5 mA; 15–16 s CBCT: 85–90 kV; 8–12.5 mA; 13.7–16 s

Radiographic interpretation was performed by an experienced radiologist blinded to biomarker data. Intra-observer reliability for lesion measurements demonstrated excellent agreement (ICC = 0.92).

### 2.4. Serum Biomarker Analysis

Blood Collection and Processing

Venous blood samples were collected under standardized conditions. After clotting (30–45 min), samples were centrifuged at 4500 rpm for 5 min at 15 °C. Serum was aliquoted and stored at −80 °C until analysis to prevent degradation and repeated freeze–thaw cycles.

ELISA Quantification

Serum Dickkopf-1 (Dkk-1) and tartrate-resistant acid phosphatase 5B (TRAP-5B) were quantified using commercial sandwich ELISA kits according to manufacturer protocols.

Dkk-1: detection range 31.25–2000 pg/mL; sensitivity <20 pg/mL; intra-/inter-assay CV <8%/<10%. TRAP-5B: detection range 0.5–20 ng/mL; sensitivity <0.1 ng/mL; intra-/inter-assay CV <10%/<12%.

All samples were analyzed in duplicate. Optical density was measured at 450 nm, and concentrations were calculated from standard calibration curves. Laboratory personnel were blinded to clinical and radiological findings.

Biomarker analysis was performed on 60 participants (30 controls and 30 patients with ENT cancer), as three samples from the cancer group were excluded due to insufficient serum volume.

The ELISA-based quantification of serum biomarkers is a widely used and validated method for assessing bone turnover and inflammatory mediators in clinical research [54,55].

### 2.5. Statistical Analysis

Prior to statistical testing, the distribution of continuous variables was assessed using the Shapiro–Wilk test to determine normality. Data are presented as mean ± standard deviation (SD) for normally distributed variables or median and interquartile range (IQR) for non-normally distributed variables. Comparisons between the cancer and control groups were performed using the independent-samples Student *t*-test for normally distributed data or the Mann–Whitney *U* test for non-normally distributed variables. Categorical variables were analyzed using the chi-square test or Fisher’s exact test, as appropriate. Correlations between oral parameters and serum biomarkers were evaluated using Pearson’s or Spearman’s correlation coefficients depending on data distribution. Effect sizes were calculated to estimate the magnitude of differences between groups. Statistical analyses were conducted using SPSS (version 27.0, IBM Corp., Armonk, NY, USA) and Statistica (version 14.0, TIBCO Software Inc., Palo Alto, CA, USA). A two-tailed *p* value ≤ 0.05 was considered statistically significant.

A priori power analysis was performed using G*Power software (version 3.1) based on a two-tailed independent-samples *t*-test. Assuming an effect size of Cohen’s d = 0.70, a significance level of α = 0.05, and a statistical power of 0.80, the minimum required sample size was calculated as 30 participants per group.

## 3. Results

### 3.1. Demographic Characteristics

The study included 63 participants (60.3% male). The control group comprised 30 individuals (15 males and 15 females), and the cancer group included 33 (N) patients (69.7% male). No significant differences were observed between groups regarding sex distribution (*p* > 0.05).

The overall mean age was 52.67 ± 12.24 years. Mean age did not differ significantly between the control group (53.87 ± 10.76 years) and the cancer group (51.58 ± 13.52 years) (*p* > 0.05).

### 3.2. Clinical Characteristics of the Cancer Group

Among patients with ENT cancer, laryngeal tumors were the most frequent (72.7%), followed by sinonasal (15.2%) and oropharyngeal neoplasms (12.1%). Radiomucositis grade 3 was observed in 60.6% of cases, and grade 4 in 39.4%, as detailed in Table 1.

Patients with laryngeal tumors were younger (49.96 ± 12.97 years) compared with those diagnosed with sinonasal tumors (58.80 ± 16.24 years).

### 3.3. Periodontal Findings

The mean number of periodontal pocket sites per patient was higher in the cancer group (0.67 ± 1.32) compared with the control group (0.37 ± 0.81); however, this difference was not statistically significant (*p* = 0.276).

When analyzed by arch, no statistically significant differences were observed for maxillary (*p* = 0.547) or mandibular (*p* = 0.312) periodontal pockets between groups. Detailed descriptive statistics are presented in Table 2.

**Figure 1:**
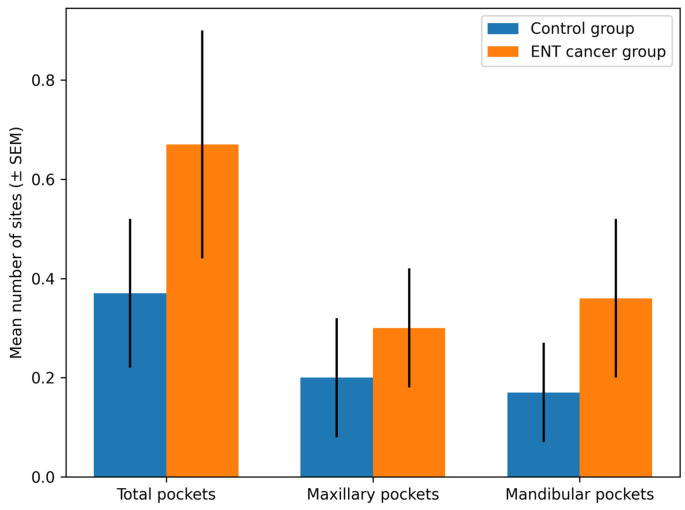
Distribution of periodontal pocket sites per patient in the control group and patients with ENT cancer.

A graphical representation of periodontal pocket distribution between the study groups is shown in Figure 1.

### 3.4. Dental Caries Assessment

#### 3.4.1. Orthopantomography (OPT)

The total number of carious lesions ranged from 0 to 8 lesions per patient. Controls exhibited a higher mean number of total caries (2.97 ± 2.48) compared with patients with ENT cancer (2.06 ± 2.29), although the difference was not statistically significant (*p* = 0.137).

No significant differences were observed between groups for maxillary or mandibular caries distribution.

Regarding maximum lesion depth, controls presented higher values (2.90 ± 1.56) compared with patients with ENT cancer (2.06 ± 1.92), without reaching statistical significance (*p* = 0.061). A significant difference was identified at the maxillary level (*p* = 0.027). Complete OPT data are summarized in Table 3.

**Figure 2:**
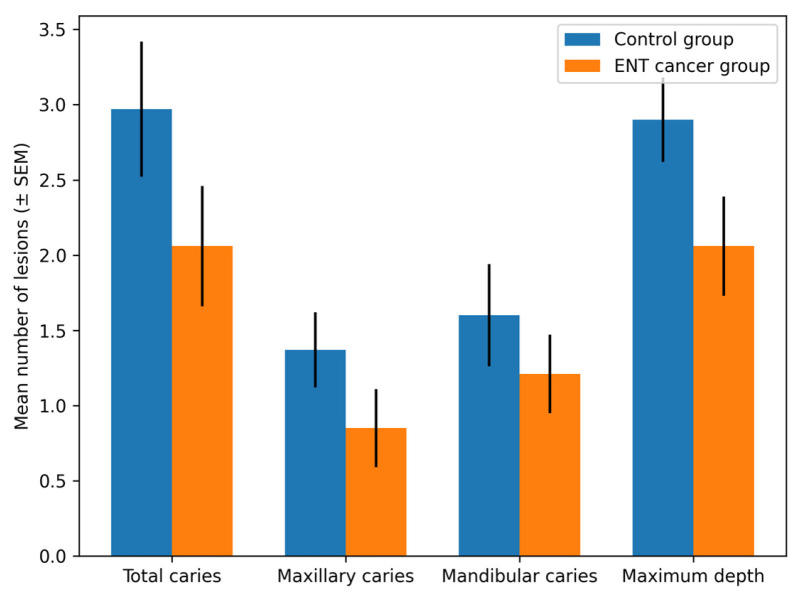
Comparison of dental caries parameters assessed by orthopantomography (OPT) between the control group and patients with ENT cancer.

A graphical representation of the OPT-assessed caries parameters is presented in Figure 2.

#### 3.4.2. Cone Beam Computed Tomography (CBCT)

CBCT analysis revealed a significantly higher mean number of total carious lesions in controls (2.97 ± 2.48) compared with patients with ENT cancer (1.85 ± 1.89) (*p* = 0.048).

Arch-specific comparisons (maxilla and mandible) did not demonstrate statistically significant differences (*p* > 0.05).

Maximum lesion depth was greater in controls (2.90 ± 1.56) than in patients with ENT cancer (2.09 ± 1.89), although this difference did not reach statistical significance (*p* = 0.068). Detailed CBCT findings are presented in Table 4.

**Figure 3:**
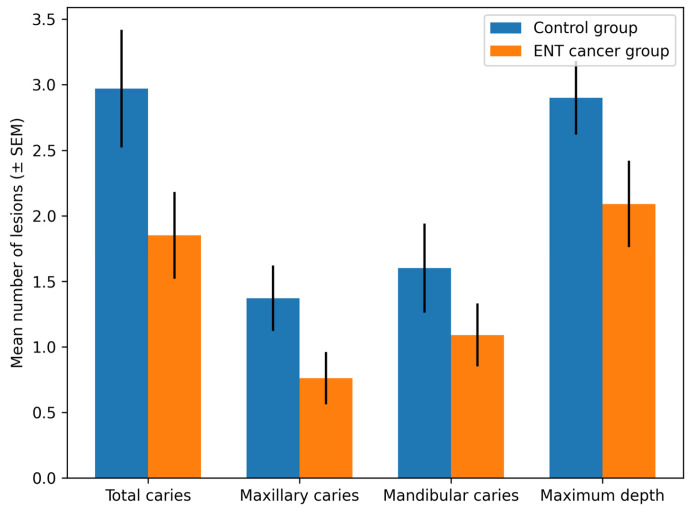
Comparison of CBCT-assessed dental caries parameters between the control group and patients with ENT cancer.

A graphical representation of the CBCT-assessed caries parameters is shown in Figure 3.

### 3.5. Serum Biomarker Levels

Significant intergroup differences were identified for both evaluated biomarkers (Table 5).

TRAP-5B levels were significantly higher in patients with ENT cancer (0.3539 ± 0.0195) compared with controls (0.3034 ± 0.0144) (t = −11.405, *p* < 0.001).

Conversely, Dkk-1 levels were significantly lower in patients with ENT cancer (1.2100 ± 0.1108) compared with controls (1.7121 ± 0.1004) (t = 18.391, *p* < 0.001).

**Figure 4:**
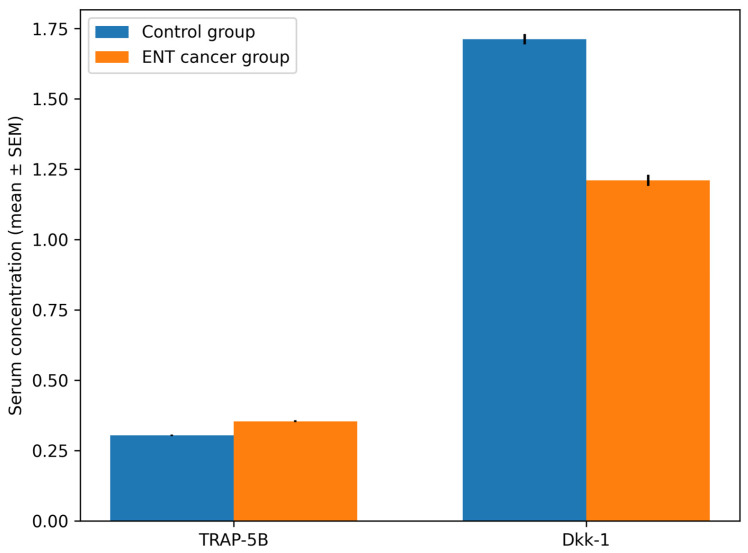
Combined comparison of serum TRAP-5B and Dkk-1 concentrations between the control group and patients with ENT cancer. TRAP-5B levels were higher in the study group, whereas Dkk-1 levels were lower compared with controls, reflecting altered bone remodeling dynamics in oncologic patients.

Graphical visualization of the biomarker data is presented in Figure 4, illustrating the distribution of TRAP-5B and Dkk-1 concentrations in the study and control groups.

### 3.6. Correlation Analysis

Correlation analysis (Table 6) revealed predominantly weak associations between serum biomarkers and oral clinical parameters in both groups. In the control group, TRAP-5B demonstrated a statistically significant moderate positive correlation with mandibular caries detected on OPT (r = 0.425, *p* < 0.05), suggesting a potential association between localized carious burden and systemic osteoclastic activity. Weak inverse correlations were also observed between TRAP-5B and periodontal pocket parameters.

In contrast, patients with ENT cancer showed generally weak and inconsistent correlations between TRAP-5B and oral parameters, indicating a possible alteration of the physiological relationship between oral inflammatory burden and systemic bone remodeling in oncologic conditions.

**Figure 5:**
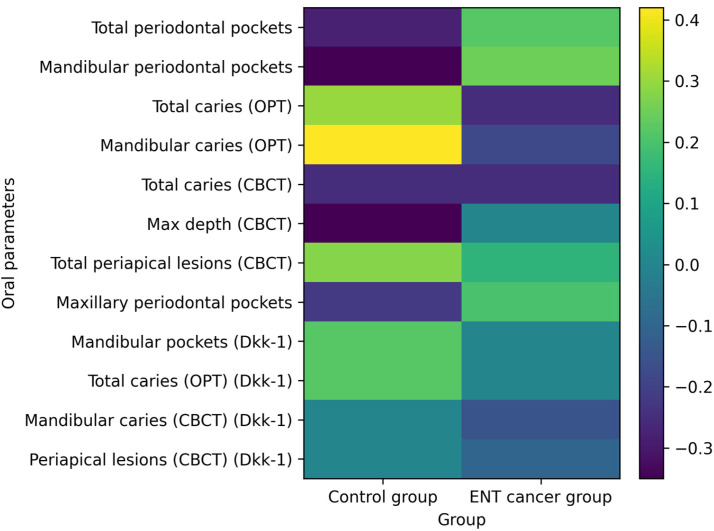
Heatmap illustrating the correlations between serum biomarkers (TRAP-5B and Dkk-1) and oral clinical parameters in the control group and patients with ENT cancer. Color intensity represents the strength and direction of the correlation coefficients (r/ρ). Most associations were weak and not statistically significant, except for a moderate positive correlation between TRAP-5B and mandibular caries detected by OPT in the control group.

Dkk-1 demonstrated weak and statistically non-significant correlations with most evaluated oral parameters in both groups, suggesting a less direct association with localized oral inflammatory activity compared with TRAP-5B. A graphical representation of these correlations is provided in Figure 5, which illustrates the overall weak correlation patterns observed between systemic biomarkers and dental or periodontal parameters.

## 4. Discussion

The present study demonstrates that patients with ENT cancer exhibit a distinct systemic bone remodeling profile characterized by significantly increased TRAP-5B levels and reduced Dkk-1 concentrations compared with healthy controls. Additionally, oncologic patients showed a higher prevalence of periodontal pockets and periapical lesions, whereas dental caries were more frequently detected in the control group. These findings are consistent with a potential cancer-associated imbalance in bone remodeling characterized by enhanced osteoclastic activity and altered Wnt/β-catenin signaling.

TRAP-5B is a well-established biomarker of osteoclast number and bone resorptive activity. The elevated levels observed in oncologic patients are consistent with increased osteoclastogenesis and tumor-related bone catabolism. Similar findings were reported by Rissanen et al. [56], who described increased osteoclastic activity in malignancy-associated bone remodeling. Moreover, TRAP-5B has been proposed as a potential marker of metastatic burden and tumor-related skeletal involvement, further supporting its clinical relevance in oncologic conditions [57].

In contrast, Dkk-1, a key inhibitor of the Wnt signaling pathway and regulator of osteoblast differentiation, was significantly reduced in patients with ENT cancer. This finding contrasts with studies reporting elevated Dkk-1 levels in inflammatory conditions, where it has been associated with increased bone resorption and disease activity. These differences suggest that Dkk-1 regulation may be context-dependent, varying between inflammatory and oncologic conditions.

In oncologic settings, Dkk-1 expression may be influenced by tumor-related factors, including cytokine signaling, tumor–bone interactions, and immune microenvironment dynamics. Previous studies have reported the involvement of Dkk-1 in tumor progression and osteolytic processes in squamous cell carcinoma [58], while its expression appears to be only partially dependent on acute inflammatory activity [59]. Therefore, the reduced Dkk-1 levels observed in the present cohort may reflect systemic tumor-mediated modulation of bone formation pathways rather than direct effects of local oral inflammation. However, given the cross-sectional design of this study, these findings should be interpreted with caution, and no causal relationships can be established.

The relationship between systemic biomarkers and oral inflammatory parameters also differed between groups. In healthy individuals, moderate correlations may reflect a physiological interaction between local inflammatory burden and systemic bone turnover. In contrast, the predominantly weak correlations observed in oncologic patients may suggest altered systemic–oral interaction patterns. Tumor-derived cytokines, systemic inflammation, and metabolic alterations associated with malignancy may influence circulating bone biomarkers and potentially reduce the impact of local oral pathology. However, these findings should be interpreted with caution given the cross-sectional design of the study.

Radiological findings obtained using orthopantomography (OPT) and cone beam computed tomography (CBCT) were consistent and supported the reliability of the imaging assessment. OPT remains a valuable tool for comprehensive evaluation of the jaws, although certain limitations in reproducibility have been reported [60,61]. In comparison, CBCT provides superior spatial resolution and improved diagnostic accuracy for detecting dental and periodontal lesions, which strengthens the validity of the imaging-based findings in the present study [62].

Recent evidence further highlights the role of Dickkopf-1 as an important mediator linking inflammation and bone remodeling. A systematic review and meta-analysis by Maharavi et al. demonstrated significantly elevated DKK1 levels in patients with periodontitis and rheumatoid arthritis, suggesting its involvement in inflammatory bone destruction and disease progression [63]. Similarly, Romero-Sánchez et al. reported correlations between serum and gingival crevicular fluid DKK1 levels and disease activity parameters in rheumatoid arthritis, supporting its potential role as a biomarker reflecting both systemic inflammation and bone loss [64].

Systemic conditions such as diabetes mellitus and smoking may further influence DKK1 expression. Miranda et al. reported increased levels of DKK1 and sclerostin in patients with chronic periodontitis associated with diabetes and smoking, suggesting synergistic effects of these conditions on inhibition of the Wnt signaling pathway and periodontal tissue destruction [65]. In addition, recent molecular studies indicate that DKK1 expression may be regulated by non-coding RNAs. Ren et al. demonstrated that the long non-coding RNA MIAT modulates DKK1 expression through the miR-204-5p axis, influencing inflammatory responses and osteogenic differentiation in periodontal ligament fibroblasts [66].

Beyond inflammatory diseases, DKK1 has also been implicated in tumor biology. Liu et al. reported increased DKK1 expression in oral squamous cell carcinoma and demonstrated associations with immune cell infiltration and patient prognosis [67]. Interestingly, higher DKK1 expression was associated with improved survival, suggesting that the biological role of this protein may vary depending on the tumor microenvironment and immune interactions.

Host-derived biomarkers in gingival crevicular fluid have also demonstrated promising diagnostic potential in periodontal disease. Biomarkers such as matrix metalloproteinase-8 (MMP-8), TRAP-5, and osteoprotegerin (OPG) have shown strong discriminatory ability between healthy, mild, and severe periodontal conditions [68]. Among these, MMP-8 demonstrated the highest diagnostic accuracy, reflecting its central role in collagen degradation and periodontal tissue destruction. OPG exhibited high specificity for severe disease due to its inhibitory effect on osteoclastogenesis, while TRAP-5 reflected active bone resorption. These findings suggest that combined biomarker panels targeting inflammation, matrix degradation, and bone turnover may provide a more comprehensive assessment of periodontal disease activity.

Several potential confounding factors should be considered when interpreting the present results. Smoking is a recognized risk factor for periodontal disease and systemic inflammation and may influence bone metabolism by promoting osteoclastic activity and impairing bone remodeling. Tumor stage may also affect systemic biomarker levels through mechanisms related to tumor burden, systemic inflammatory responses, and tumor-induced osteolysis. Although stratified analyses according to smoking status or tumor stage were not performed in the present study, these factors should be addressed in future investigations.

Overall, the findings of this study are consistent with the possibility that malignancy may be associated with systemic dysregulation of bone remodeling characterized by increased osteoclast activity and altered modulation of the Wnt signaling pathway. Under such conditions, the influence of local oral inflammatory lesions on circulating bone biomarkers appears to be secondary to tumor-related systemic effects.

### 4.1. Clinical and Biological Implications

The findings of the present study highlight the potential relevance of systemic bone turnover biomarkers in understanding oral–systemic interactions in oncologic patients. The elevated TRAP-5B levels observed in the cancer group suggest increased osteoclastic activity, which may reflect tumor-associated alterations in bone metabolism. Conversely, the reduced Dkk-1 concentrations may indicate dysregulation of the Wnt/β-catenin signaling pathway, a key regulator of bone formation and remodeling. These alterations appear to occur largely independently of the local oral inflammatory burden, as demonstrated by the generally weak correlations between serum biomarkers and oral clinical parameters.

From a clinical perspective, these results emphasize the importance of comprehensive oral assessment in patients with head and neck cancer, particularly prior to the initiation of oncologic therapy. Early identification and management of oral inflammatory lesions may contribute to reducing treatment-related complications and improving overall patient outcomes within a multidisciplinary care framework.

Furthermore, the observed dissociation between local oral pathology and systemic bone turnover markers suggests that the interpretation of TRAP-5B and Dkk-1 in oncologic patients should consider the influence of systemic disease processes. In this context, these biomarkers may reflect broader tumor–bone interactions rather than localized inflammatory activity alone, with potential implications for risk stratification, monitoring of skeletal involvement, and multidisciplinary management of patients undergoing cancer therapy.

### 4.2. Study Limitations

This study is limited by its cross-sectional design, which precludes causal inference. The relatively small sample size may have reduced the statistical power to detect moderate associations. Biomarkers were assessed at a single time point, and longitudinal dynamics were not evaluated. Additionally, cancer stage, therapeutic exposure, and systemic inflammatory indices were not stratified. Although both OPT and CBCT were used, early subclinical lesions may remain radiographically undetected.

Future prospective studies with larger cohorts and longitudinal biomarker assessment are required to clarify the temporal relationship between tumor progression, systemic bone remodeling, and oral inflammatory burden.

## 5. Conclusions

The present study demonstrates that patients with ENT cancer exhibit a significantly altered systemic bone remodeling profile, characterized by increased TRAP-5B levels and decreased Dkk-1 concentrations compared with healthy controls. These findings suggest an imbalance in bone turnover associated with oncologic conditions.

Although oral inflammatory lesions, including periodontal pockets and periapical lesions, were more prevalent in patients with ENT cancer, their correlation with systemic biomarkers was generally weak. This may suggest altered systemic–oral interaction patterns in oncologic patients.

Overall, these results highlight the importance of integrated clinical, radiological, and biochemical assessments in patients with head and neck cancer, while emphasizing the need for further longitudinal studies to clarify the underlying mechanisms.

## Figures and Tables

**Table 1:** Distribution of cancer types and radiomucositis grades in the study group (n = 33).

Cancer Type	n	% (N)	Radiomucositis Grade	n	% (N)
Laryngeal neoplasm	24	72.7	Grade 3	20	60.6
Oropharyngeal neoplasm	4	12.1	Grade 4	13	39.4
Sinonasal neoplasm	5	15.2	—	—	—
Total	33	100	Total	33	100

**Table 2:** Comparative descriptive statistics of periodontal pocket distribution between the control and cancer groups.

Variable	Group	n	Mean	SEM	SD	Min	Max	*t* Value	*p* Value
Total periodontal pockets	Control	30	0.37	0.15	0.81	0	3	−1.101	0.276
	Cancer	33	0.67	0.23	1.32	0	5		
	Total	63	0.52	0.14	1.11	0	5		
Maxillary periodontal pockets	Control	30	0.20	0.12	0.66	0	3	−0.605	0.547
	Cancer	33	0.30	0.12	0.68	0	2		
	Total	63	0.25	0.09	0.67	0	3		
Mandibular periodontal pockets	Control	30	0.17	0.10	0.53	0	2	−1.019	0.312
	Cancer	33	0.36	0.16	0.93	0	4		
	Total	63	0.27	0.10	0.77	0	4		

Values are expressed as mean ± standard error of the mean (SEM). Statistical analysis was performed using the independent-samples Student *t*-test. SEM—standard error of the mean; SD—standard deviation.

**Table 3:** Dental caries (OPT exam) descriptive statistics (control group, study group).

Parameter	Group	N	Mean	SEM	SD	Min	Max	Median	t (Student)	*p*
Total dental caries (OPT)—number	Control	30	2.97	0.454	2.484	0	8	2.00	1.506	0.137
	Study	33	2.06	0.399	2.290	0	8	1.00		
	Total	63	2.49	0.303	2.409	0	8	2.00		
Maxillary dental caries (OPT)—number	Control	30	1.37	0.251	1.377	0	4	1.00	1.422	0.160
	Study	33	0.85	0.262	1.503	0	6	0.00		
	Total	63	1.10	0.183	1.456	0	6	0.00		
Mandibular dental caries (OPT)—number	Control	30	1.60	0.341	1.868	0	6	1.00	0.909	0.367
	Study	33	1.21	0.264	1.516	0	5	1.00		
	Total	63	1.40	0.213	1.690	0	6	1.00		
Total dental caries (OPT)—maximum depth	Control	30	2.90	0.285	1.561	0	4	4.00	1.911	0.061
	Study	33	2.06	0.334	1.919	0	4	2.00		
	Total	63	2.46	0.226	1.794	0	4	4.00		
Maxillary dental caries (OPT)—maximum depth	Control	30	2.13	0.328	1.795	0	4	3.00	2.268	0.027 *
	Study	33	1.15	0.286	1.642	0	4	0.00		
	Total	63	1.62	0.223	1.773	0	4	0.00		
Mandibular dental caries (OPT)—maximum depth	Control	30	2.03	0.341	1.866	0	4	2.50	0.719	0.475
	Study	33	1.70	0.321	1.845	0	4	1.00		
	Total	63	1.86	0.233	1.848	0	4	1.00		

Values are expressed as mean ± standard error of the mean (SEM). Statistical analysis was performed using the independent-samples Student *t*-test. SEM—standard error of the mean; SD—standard deviation. * Statistically significant at *p* < 0.05.

**Table 4:** Descriptive statistics of dental caries assessed by CBCT examination in the control and study groups.

Variable	Group	N	Mean	SEM	SD	Min	Max	Median	t (Student)	*p*-Value
Total dental caries (CBCT)—no.	Control	30	2.97	0.454	2.484	0	8	2.00	2.022	0.048 *
	Study	33	1.85	0.329	1.889	0	6	2.00		
	Total	63	2.38	0.283	2.246	0	8	2.00		
Maxillary dental caries (CBCT)—no.	Control	30	1.37	0.251	1.377	0	4	1.00	1.935	0.058
	Study	33	0.76	0.195	1.119	0	4	0.00		
	Total	63	1.05	0.161	1.275	0	4	1.00		
Mandibular dental caries (CBCT)—no.	Control	30	1.60	0.341	1.868	0	6	1.00	1.246	0.217
	Study	33	1.09	0.236	1.355	0	5	1.00		
	Total	63	1.33	0.205	1.626	0	6	1.00		
Total dental caries (CBCT)—maximum depth	Control	30	2.90	0.285	1.561	0	4	4.00	1.857	0.068
	Study	33	2.09	0.330	1.893	0	4	2.00		
	Total	63	2.48	0.224	1.777	0	4	4.00		
Maxillary dental caries (CBCT)—maximum depth	Control	30	2.13	0.328	1.795	0	4	3.00	1.954	0.055
	Study	33	1.27	0.296	1.701	0	4	0.00		
	Total	63	1.68	0.225	1.785	0	4	1.00		
Mandibular dental caries (CBCT)—maximum depth	Control	30	2.03	0.341	1.866	0	4	2.50	0.859	0.394
	Study	33	1.64	0.313	1.800	0	4	1.00		
	Total	63	1.83	0.230	1.828	0	4	1.00		

Values are expressed as mean ± standard error of the mean (SEM). Statistical analysis was performed using the independent-samples Student *t*-test. SEM—standard error of the mean; SD—standard deviation. * Statistically significant at *p* < 0.05.

**Table 5:** Comparative descriptive statistics of ELISA kit biomarkers in the control and study groups.

Biomarker	Group	N	Mean	SEM	SD	Min	Max	t (Student)	*p*-Value
TRAP-5b	Control	30	0.3034	0.00263	0.01441	0.26935	0.33186	−11.405	<0.001 **
	Study	30	0.3539	0.00357	0.01953	0.32018	0.39184		
	Total	60	0.3287	0.00396	0.03064	0.26935	0.39184		
Dkk-1	Control	30	1.7121	0.01833	0.10040	1.30837	1.84554	18.391	<0.001 **
	Study	30	1.2100	0.02023	0.11083	0.92538	1.38710		
	Total	60	1.4610	0.03538	0.27403	0.92538	1.84554		

Values are expressed as mean ± standard error of the mean (SEM). Statistical analysis was performed using the independent-samples Student *t*-test. SEM—standard error of the mean. SD—standard deviation. ** Statistically significant at *p* < 0.001.

**Table 6:** Correlation between Serum Biomarkers and Oral Parameters in Control and Cancer Groups.

Biomarker	Oral Parameter	Control (r/ρ; *p*)	Effect Size	Cancer (r/ρ; *p*)	Effect Size
TRAP-5B	Total periodontal pockets	−0.302; *p* > 0.05	Weak	0.189; *p* > 0.05	Weak
	Mandibular periodontal pockets	−0.360; *p* > 0.05	Weak–Moderate	0.241; *p* > 0.05	Weak
	Total caries (OPT)	0.285; *p* > 0.05	Weak	−0.272; *p* > 0.05	Weak
	Mandibular caries (OPT)	0.425; *p* < 0.05	Moderate	−0.189; *p* > 0.05	Weak
	Total caries (CBCT)	−0.269; *p* > 0.05	Weak	−0.269; *p* > 0.05	Weak
	Maximum caries depth (CBCT)	−0.357; *p* > 0.05	Weak–Moderate	NS	—
	Total periapical lesions (CBCT)	0.271; *p* > 0.05	Weak	0.137; *p* > 0.05	Weak
Dkk-1	Total periodontal pockets	NS	—	0.103; *p* > 0.05	Weak
	Maxillary periodontal pockets	−0.248; *p* > 0.05	Weak	0.203; *p* > 0.05	Weak
	Mandibular periodontal pockets	0.193; *p* > 0.05	Weak	NS	—
	Total caries (OPT)	0.204; *p* > 0.05	Weak	NS	—
	Mandibular caries (CBCT)	NS	—	−0.150; *p* > 0.05	Weak
	Total periapical lesions (CBCT)	NS	—	−0.109; *p* > 0.05	Weak

Abbreviations: r—Pearson correlation coefficient; ρ—Spearman correlation coefficient; NS—not statistically significant (*p* > 0.05). Effect size interpretation: |r| < 0.30 = weak; 0.30–0.49 = moderate; ≥0.50 = strong.

## Data Availability

All the data used in this study are available on request from the corresponding author.
